# Acute paraparesis syndrome after ruptured anterior communicating artery aneurysm

**DOI:** 10.1097/MD.0000000000028792

**Published:** 2022-02-04

**Authors:** Jong-Myong Lee

**Affiliations:** Department of Neurosurgery, Jeonbuk National University Hospital and Medical School, Jeon-Ju, Republic of Korea.

**Keywords:** cerebral edema, hydrocephalus, paraparesis, subarachnoid hemorrhage

## Abstract

Here, we describe a series of 7 patients who presented with acute paraparesis due to anterior communicating artery aneurysm rupture. This study aimed to assess the clinical and radiological factors associated with acute paraparesis syndrome caused by subarachnoid hemorrhage (SAH).

Between June 2005 and December 2012, our institution consecutively treated 210 patients with anterior communicating aneurysm rupture within 24 hours after ictus. We divided the patients into 2 groups based on the presence (n = 7) and absence (n = 203) of acute paraparesis after anterior communicating aneurysm rupture.

Diffusion-weighted magnetic resonance imaging revealed high intensity in the medial aspects of the bilateral frontal lobes in 3 patients. The mean third ventricular distance at the time of admission was 9.2 mm (range, 8–12.5 mm), and the mean bicaudate distance was 33.9 mm (range, 24–39 mm). There was a significant difference in the bicaudate distance (*P* = .001) and third ventricle distance (*P* = .001) between the 2 groups. Acute hydrocephalus and global cerebral edema (GCE) were confirmed radiologically in all patients in the acute paraparesis group. The presence of acute hydrocephalus (*P* = .001) and GCE (*P* = .003) were significantly different between the groups.

Acute paraparesis syndrome after SAH is transient and gradually improves if the patient does not develop severe vasospasm. The present study demonstrates that acute paraparesis after SAH is associated with acute hydrocephalus and GCE.

## 1 Introduction

Subarachnoid hemorrhage (SAH) should always be suspected in patients with a typical presentation, including a sudden onset of severe headache accompanied by nausea, vomiting, neck pain, photophobia, and loss of consciousness.^[1]^ Patients with aneurysmal SAH and acute focal neurological deficits are at high risk of developing permanent neurological deficits.^[2]^ Major causes of acute focal neurological deficits are primary brain damage due to space-occupying hematomas or immediate postsurgical complications such as vessel clip occlusion, thromboembolic events, or early brain swelling.^[2]^ During aneurysmal SAH, intracranial pressure (ICP) increases, resulting in a sharp decrease in cerebral perfusion pressure (CPP).^[3]^ Subsequent to SAH, many survivors experience constriction of the cerebral arteries, commonly described as vasospasm. Arterial constrictions typically occur within 3 to 21 days after SAH and may last for 12 to 16 days.^[4–7]^ Seven days after SAH, angiographic evidence indicating vascular constriction, commonly termed “arterial vasospasm” is present in approximately 30% to 70% of patients, and neurologic deficits due to cerebral infarction are usually seen in around 30% of patients during the vasospasm periods.^[4]^ Focal neurologic deficits occur in a delayed fashion several days after SAH.

The incidence of acute paraparesis within 24 hours after SAH is very rare, and the pathogenesis of these symptoms remains poorly defined. We evaluated the clinical and radiologic findings in 7 patients with acute paraparesis syndrome within 24 hours after ictus. This study aimed to assess the clinical and radiologic findings of acute paraparesis syndrome caused by SAH and its clinical outcomes.

## 2 Materials and methods

### 2.1 Patient population and clinical features

We reviewed the clinical and radiological information of all patients admitted to our institution with acute aneurysmal SAH between January 2005 and December 2012. The study protocol was approved by our institutional review board. Patients with traumatic and mycotic aneurysms were excluded from the study. Patients with lesions of the spinal cord and intracranial lesions associated with paraparesis, such as brain tumors, head injuries, cerebral infarction, and venous sinus thrombosis were also excluded. The inclusion criteria were as follows: angiographically confirmed saccular aneurysm, onset of SAH within 24 hours before admission, paraparesis developed with SAH within 24 hours after ictus, and uneventful surgery. Paraparesis was identified based on responses to verbal orders or pain in patients with a disturbance of consciousness. The degree of paraparesis was assessed using the Medical Research Council (MRC) scale at the time of admission. The diagnosis of SAH was based on positive computed tomography (CT) scans. The clinical condition at admission was assessed using the Hunt and Hess (H-H) grades. The collected data included general demographic information, history of hypertension, smoking, diabetes mellitus, onset of SAH, radiological Fisher grade, location and size of the aneurysm, presence of acute hydrocephalus, treatment modality chosen to secure the aneurysm (surgical clipping or endovascular coil occlusion), and timing of treatment in relation to SAH onset.

Global cerebral edema (GCE) was diagnosed when both of the following were present: complete or near-complete effacement of the hemispheric sulci and basal cisterns and bilateral and extensive disruption of the hemispheric gray-white matter junction at the level of the centrum semiovale, which was due to either blurring or a diffuse peripheral “fingerlike” extension of the normal demarcation between gray and white matter.^[4,8–12]^ At our institution, pre-operative ventriculostomy is not routinely performed in patients with cerebral edema. In patients with GCE, we initially performed a large craniotomy and cisternal drainage of cerebrospinal fluid. If we failed to obtain brain slackness despite these procedures, we performed a ventriculostomy.

Ventricular enlargement on the admission CT scan was quantified by measuring the bicaudate index (BCI) and the width of the third ventricle. Brain CT scans obtained using a GE Light Speed Ultra CT scanner in 5-mm axial slices were reviewed. The BCI is the width of the frontal horns at the level of the caudate nuclei and the foramen of Monro divided by the corresponding diameter of the brain.^[13,14]^ To calculate age-adjusted relative sizes, the relative BCIs were divided by the corresponding upper limit (95th percentile) per age group. The widest diameter of the third ventricle (mm) was measured. Acute hydrocephalus was defined as a relative BCI greater than 1.

Symptomatic vasospasm was defined as a documented arterial vasospasm consistent with new neurological deficits presenting within 21 days after the onset of SAH and not explained by other causes of neurological deterioration (re-bleeding, acute or worsening hydrocephalus, electrolyte disturbances, hypoxia, or seizures).^[7,15,16]^ All focal deficits were ascribed to the vascular territory, which could best explain the symptoms. We divided the patients into 2 groups based on the presence (n = 7) or absence (n = 203) of acute paraparesis after anterior communicating aneurysm rupture. The patients’ pre-operative H-H scale at presentation, age, Fisher grade, time of operation, aneurysm size, as well as their neurological status, clinical course, and outcome were compared between the groups. The H-H grading system scale at presentation and the age at diagnosis were used because they are accepted prognostic factors for this disease. Clinical outcomes were assessed using the Glasgow Outcome Scale (GOS) at discharge and at the last clinical follow-up visit. Clinical status at the last follow-up visit was defined as the final outcome. For ease of analysis and reporting, the 5-point GOS score was categorized as either favorable (moderate disability or good recovery) or unfavorable (dead, vegetative, or severe disability).

### 2.2 Statistical analysis

Survival and functional outcomes at discharge and at 3 months were assessed using GOS. To assess the validity of this approach, we performed a confirmatory univariate analysis using chi-squared tests for categorical variables, two-tailed *t* tests for normally distributed continuous variables, and Mann-Whitney *U* tests for non-normally distributed continuous variables. The Mann-Whitney test was used for non-categorical variables (age, bicaudate distance, and third ventricle diameter). The following variables were tested by univariate logistic regression analysis: sex, presence of comorbidities (hypertension, smoking, diabetes mellitus), pre-operative acute hydrocephalus, cerebral edema, bicaudate distance, third ventricle distance, and clinical outcome. A logistic regression analysis with a forward stepwise method was then performed to assess the independent association of acute paraparesis after SAH with other clinical and radiologic factors. Statistical analyses were performed using the SPSS/PC+ statistical package (SPSS Inc., Chicago, IL).

## 3 Results

### 3.1 Patient characteristics and outcome

**Figure 1:**
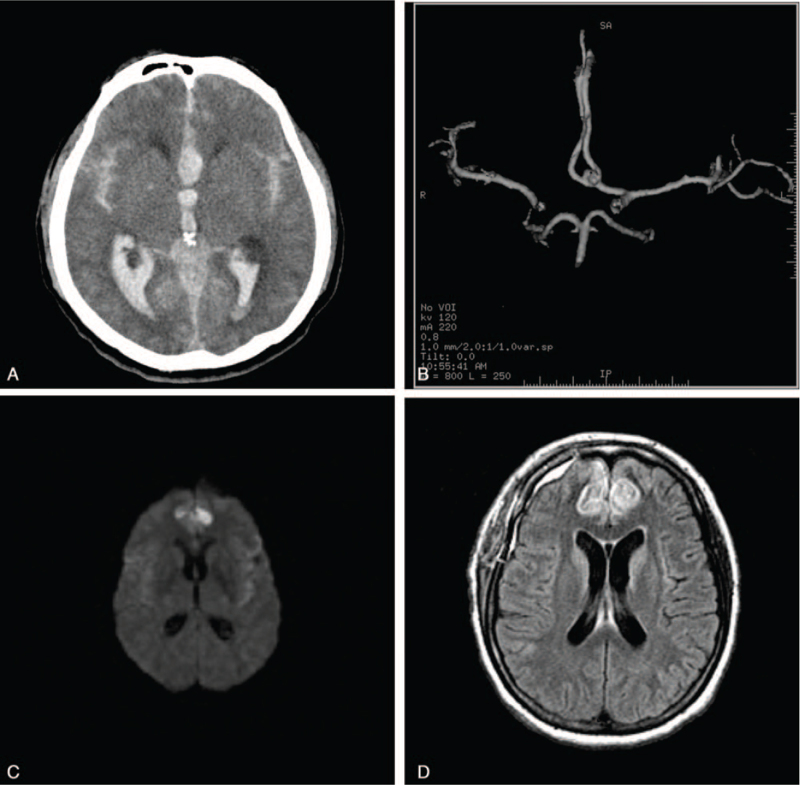
(A) Brain CT scans on admission showed a thick subarachnoid hemorrhage and global edema. (B) CTA revealed an anterior communicating artery aneurysm. (C) Postoperative diffusion-weighted imaging showed a high signal intensity area in the medial aspect of the bilateral frontal lobes. (D) Postoperative FLAIR image showing a high signal intensity area in the medial aspect of the frontal lobes. CT = computed tomography, CTA = computed tomographic angiography, FLAIR = fluid attenuated inversion recovery sequence.

The degree of paraparesis and the baseline characteristics are summarized in Table 1. Neurological symptoms of paraparesis occurred within 24 hours after ictus in all patients. The clinical follow-up duration was a mean of 44.7 months. The median age was 52.3 years (range, 42–65 years), and 4 patients were women. Three patients had a history of hypertension and 2 patients had diabetes mellitus. There were no significant differences in age, sex, smoking, or past medical history of hypertension and diabetes mellitus between the 2 groups (Table 2). There was no evidence of aneurysmal re-bleeding located in the anterior communicating artery aneurysm in any of the patients (0/7 patients) (Fig. 1A and B). The mean aneurysm size was 6 mm (range, 3–10 mm). Six patients (91%) underwent surgical clipping, and 1 patient (9%) was treated with endovascular coil embolization. The incidence of vasospasm was 71% (5/7) in the paraparesis group and 26.6% (54/203) in the control group. There was a statistically significant difference in the incidence of vasospasm between the groups (*P* = .030) (Table 2).

**Table 1:** Baseline patient characteristics of acute paraparesis syndrome.

Patients no.	Age	H-H Gr	Fisher Gr	Focal neurologic sign	3^rd^ VD (mm)	BCD (mm)	HDC	Edema	Motor Gr	GOS	Vasospasm	Recovery time	Recovery Motor Gr
1	42/F	III	III	No	6	24	Yes	Yes	III/III	SD	Yes	NA	NA
2	52/F	IV	III	No	8	34	Yes	Yes	II/II	GR	Yes	3Mo	V/V
3	46/M	IV	IV	No	5	34	Yes	Yes	II/III	MD	Yes	7D	V/V
4	65/F	IV	IV	No	8	33	Yes	Yes	II/II	GR	Yes	8D	V/V
5	60/F	III	III	No	7.5	35	Yes	Yes	III/III	GR	No	1D	V/V
6	47/M	IV	IV	No	12	39	Yes	Yes	I/III	GR	Yes	3D	V/V
7	54/M	III	IV	No	12.5	38	Yes	Yes	III/III	GR	No	5D	V/V

3^rd^ VD = 3^rd^ ventricle distance, BCD = bicaudate distance, D = day, Edema = global cerebral edema, F = female, Fisher Gr = Fisher grade, GOS = Glasgow Outcome Scale, GR = good recovery, HDC = hydrocephalus, H-H Gr = Hunt and Hess grade, M = male, MD = moderate disability, Mo = month, Motor Gr = initial lower extremity Medical Research Council grade, NA = not applicable, SD = severe disability.

**Table 2:** Summary of patient data by group.

	Paraparesis group (N = 7)	Control group (N = 203)	*P* value	Univariate regression
Age (yrs)	52.3	51.5	*P* = .929^∗^	
Sex (M/F)	(3/4)	(105/98)	*P* = .468^†^	
GCS (mean)	9.29	12.88	*P* = .001^∗^	
An size (mm)	5.86	5.96	*P* = .763^∗^	
H-H Gr (II/III/IV/V)	(0/3/4/0)	(89/84/17/13)	*P* = .050^†^	
Fisher Gr (I/II/III/IV)	(0/3/4/0)	(7/108/107/71)	*P* = .170^†^	
3^rd^ ventricle distance	9.2 mm	6.9 mm	*P* = .001^∗^	
Bicaudate distance	33.9 mm	24.9 mm	*P* = .001^∗^	
Hydrocephalus (n)	7 (100%)	33 (16.25%)	*P* = .001^†^	*P* < .001
GCE (n)	7 (100%)	41 (20.2%)	*P* = .003^†^	*P* < .001
Clinical vasospasm (n)	5 (71%)	54 (26.6%)	*P* = .020^†^	*P* = .037
Hypertension (n)	3 (43%)	71 (34.9%)	*P* = .294^†^	
DM (n)	2 (28.5%)	17 (8.4%)	*P* = .213^†^	
Smoking (n)	2 (28.5%)	51 (25.1%)	*P* = .681^†^	
GOS (GR/MD/SD/D)	5/1/1/0	164/22/11/6	*P* = .124^†^	

DM = diabetics mellitus, Fisher Gr = Fisher grade, GCE = global cerebral edema, GCS = Glasgow Coma Scale, GOS = Glasgow Outcome Scale, H-H Gr = Hunt and Hess grade.

∗ Mann-Whitney test.

† Fisher exact test.

In the paraparesis group, 3 patients were H-H grade III and 4 patients were grade IV, while the Fisher grade was grade III in 3 patients and grade IV in 4 patients. The H-H and Fisher grades of the control group are summarized in Table 2. The mean Glasgow Coma Scale (GCS) was 9.29 in the paraparesis and 12.88 in the control groups. Acute paraparesis syndrome was associated with pre-operative H-H grade (*P* = .05) and pre-operative GCS score (*P* = .001) (Table 2).

At the final follow-up evaluation, the clinical outcomes included good recovery in 5 of 7 patients, moderate disability in 1 patient, and severe disability in 1 patient. In the control group, the clinical outcomes were good recovery in 164 of 203 patients, moderate disability in 22, severe disability in 11, and death in 16. There was no significant difference in the clinical outcomes between the groups (*P* = .124) (Table 2). Acute paraparesis was transient and almost completely resolved in 6 patients who could walk at discharge. One patient who did not recover from paraparesis sustained severe brain damage during the course of the disease. Three patients had cognitive and affective manifestations with a progressive resolution of symptoms. One patient had a severe personality change and underwent neuropsychiatric consultation.

### 3.2 Radiologic results

Five of the 7 patients underwent diffusion-weighted magnetic resonance image (MRI). Diffusion-weighted MRI revealed high intensity in the medial aspects of the bilateral frontal lobes in 5 patients (Fig. 1C and D). In the paraparesis group, the mean third ventricular distance at the time of admission was 9.2 mm (range, 8–12.5 mm), and the mean bicaudate distance was 33.9 mm (range, 24–39 mm). However, the third ventricle diameter was 6.9 mm (range, 4–10 mm), and the bicaudate distance was 24.9 mm (range, 23–34 mm) in the control group. There was a significant difference in the bicaudate distance (*P* = .001) and third ventricle distance (*P* = .001) between the 2 groups (Table 2). In the paraparesis group, radiologic acute hydrocephalus and GCE were confirmed in all patients (Fig. 1A). In the control group, radiologic hydrocephalus was confirmed in 33 of 203 patients (16.25%), and GCE was noted in 41 patients (20.2%). Acute paraparesis syndrome after SAH was associated with acute hydrocephalus (*P* = .001) and global edema (*P* = .003) (Table 2). Acute paraparesis syndrome after SAH occurs frequently in patients with acute hydrocephalus and global edema. Two radiologic variables were associated with acute paraparesis syndrome on univariate analysis. Forward stepwise logistic regression identified GCE (*P* < .001) and acute hydrocephalus (*P* < .001) as independent predictors of acute paraparesis syndrome (Table 2).

## 4 Discussion

This study demonstrates that acute paraparesis syndrome after SAH is associated with acute hydrocephalus and GCE. Our series has some similarities to previously published reports, such as the presence of hydrocephalus, and the time course and overall recovery rate of lower extremity function, yet distinct differences have also been identified.^[1,17,18]^ We found that acute paraparesis after SAH was more prevalent in patients with poor clinical status, GCE, and hydrocephalus.^[13]^ In our series of consecutive patients with aneurysmal paraparesis syndrome, we identified 3 common clinical characteristics: radiological acute hydrocephalus, GCE, and onset within 24 hours after ictus.

### 4.1 Global cerebral edema

GCEs after SAH are associated with brain metabolic distress.^[5,12,19]^ The pathophysiological mechanisms are incompletely understood. Rapid increases in ICP and brain circulatory arrest in the initial minutes after the onset of SAH are followed by a lasting reduction in cerebral blood flow (CBF) and may result in cytotoxic cerebral edema.^[5,6,11,20–22]^ The combination of substrate deficiency and cellular (mitochondrial) dysfunction results in brain metabolic distress, indicated by markers of anaerobic metabolism.^[9,23]^ Several mechanisms may lead to the development of acute paraparesis syndrome. No prior study has focused on GCE as a cause of paraparesis after SAH.

GCE was observed on the admission brain CT scan in 6% of patients in the International Cooperative Study on the Timing of Aneurysm Surgery.^[24]^ The rapid increase in ICP and brain circulatory arrest in the minutes initially following the onset of SAH is followed by a lasting reduction in CBF, which may result in cytotoxic cerebral edema.^[5,8,12,25,26]^ It results in brain metabolic distress, and vasogenic edema may occur when breakdown of the brain-blood barrier occurs.^[5,12,25]^ Elevated ICP in patients with global brain edema has been identified after SAH and traumatic brain injury, and has been proposed to be a general reflex phenomenon after severe brain injury.^[5,27,28]^ Secondary reductions are caused by independent factors such as vasoconstriction.^[5,10,16,28]^ Cerebral arteries have been observed to react to SAH in a biphasic pattern, with acute vasoconstriction starting immediately after SAH and delayed vasospasm occurring several days after the insult.^[10,15,27,29,30]^ Acute ischemia from SAH has been attributed to CPP, and this is supported by data from repeat hemorrhages in humans with ICP monitors.^[8,12,15,22,30]^ It is possible that cerebral circulatory arrest caused by high ICP during bleeding results in global ischemic brain injury. It also seems likely that the initial impact, including cytotoxic and vasogenic edema, may increase the cerebral energy demand during the early recovery phase after SAH.^[8,12]^ The impact of GCE is more global than that of local edema. GCE showed microvascular injury after SAH, and decreased CPP by GCE can lead to transient diffuse ischemia. Transient diffuse ischemia may lead to acute paraparesis syndrome. Our study revealed that radiologic GCE was confirmed in all patients, and acute paraparesis syndrome after SAH occurred frequently in patients with GCE.

### 4.2 Acute hydrocephalus after SAH

Johnston's study revealed that all patients with acute paraparesis had acute hydrocephalus identified on admission brain CT.^[1]^ Our study also showed that acute hydrocephalus was confirmed in all patients. It is plausible that acute hydrocephalus has a negative influence on cerebral perfusion, and acute hydrocephalus on the admission scan may be a risk factor for cerebral ischemia. Acute hydrocephalus often leads to clinical deterioration, and spontaneous recovery occurs within 24 hours.^[13,14,31,32]^ CBF in acute hydrocephalus is reduced in the basal ganglia and periventricular white matter, but not in the cortex.^[13]^ Assaf et al^[33]^ suggested that the impact of acute hydrocephalus is more local than that of the global process. Some authors have revealed that fractional anisotropy is increased in the white matter areas lateral to the ventricles in acute hydrocephalus.^[34,35]^ The increased volume of the CSF in hydrocephalus frequently leads to pressure on the most adjacent white fiber pathways: the corona radiata and the corpus callosum.^[34–36]^ Acute hydrocephalus frequently leads to pressure on the most adjacent white fiber pathways and the internal capsule.^[33,35]^ Diffusion tensor images in patients with hydrocephalus showed that the morphology of the internal capsule changed under mechanical pressure.^[33]^ Some authors have suggested that acute hydrocephalus may be a key factor, and structural deformity of white matter tracks may result in marked dysfunction of fiber tracks, producing significant paraparesis.^[1]^ Hayashi et al^[37]^ demonstrated that CBF was reduced in the hemispheric gray matter in patients with acute hydrocephalus after SAH. Cerebral perfusion on admission is a risk factor for the development of cerebral ischemia, and the absence of a relationship between acute hydrocephalus and cerebral ischemia further suggests that factors other than hydrocephalus determine cerebral perfusion on admission. Although ICP was not measured in our study, data from previous studies indicate that the initial ICP elevation after hydrocephalus induction would be sufficient to cause cerebrovascular compression, resulting in a decrease in CBF during short-term hydrocephalus stages.^[28]^ Acute hydrocephalus causes a local decrease in CBF instead of more generalized effects and leads to pressure on the most adjacent white fiber pathways and internal capsule. Our study showed that acute paraparesis syndrome is transient and gradually improves, acute hydrocephalus lead to reversible and localized effect on the most adjacent structures

In our series, there was no significant difference in the clinical outcomes between the 2 groups (*P* = .124). However, univariate Cox analysis confirmed that pre-operative GCS score (*P* = .001) and presence of vasospasm (*P* = .021) were predictors of unfavorable clinical outcomes. This may be related to selection bias due to the small sample size and the simplicity of the outcome grading system (favorable and unfavorable outcomes).

Our study has several limitations. First, this study is a retrospective study, and therefore may have sources of bias and variation. Further study is required to clarify association between factors and paraparesis syndrome. Second, the sample of 7 patients was relatively small. Third, pre-operative MRI performed in only 4 patients, we could not analyze the MRI finding in all patients. A prospective study with a larger number of cases is needed to confirm the association of acute paraparesis syndrome to clinical and radiologic factors.

## 5 Conclusions

Acute paraparesis syndrome after SAH is transient and gradually improves if the patient does not develop severe vasospasm. The present study demonstrates that acute paraparesis after SAH is associated with acute hydrocephalus and GCE. Further research is needed to elucidate the pathogenesis of this disorder, apply imaging strategies that can quantify brain water content and cerebral perfusion, and develop intensive care management strategies that can prevent or minimize brain edema after SAH.

## Author contributions

**Conceptualization:** Jong Myong Lee.

**Data curation:** Jong Myong Lee.

**Formal analysis:** Jong Myong Lee.

**Investigation:** Jong Myong Lee.

**Methodology:** Jong Myong Lee.

**Project administration:** Jong Myong Lee.

**Resources:** Jong Myong Lee.

**Writing – original draft:** Jong Myong Lee.

**Writing – review & editing:** Jong Myong Lee.
